# Dysregulated MicroRNA Involvement in Multiple Sclerosis by Induction of T Helper 17 Cell Differentiation

**DOI:** 10.3389/fimmu.2018.01256

**Published:** 2018-06-04

**Authors:** Chen Chen, Yifan Zhou, Jingqi Wang, Yaping Yan, Lisheng Peng, Wei Qiu

**Affiliations:** ^1^Department of Neurology, The Third Affiliated Hospital of Sun Yat-sen University, Guangzhou, China; ^2^Key Laboratory of the Ministry of Education for Medicinal Resources and Natural Pharmaceutical Chemistry, National Engineering Laboratory for Resource Development of Endangered Crude Drugs in Northwest of China, College of Life Sciences, Shaanxi Normal University, Xi’an, China

**Keywords:** microRNA, T helper 17 cells, multiple sclerosis, treatment, experimental autoimmune encephalomyelitis

## Abstract

Multiple sclerosis (MS) is an immune-mediated demyelinating disease of the central nervous system. Growing evidence has proven that T helper 17 (Th17) cells are one of the regulators of neuroinflammation mechanisms in MS disease. Researchers have demonstrated that some microRNAs (miRNAs) are associated with disease activity and duration, even with different MS patterns. miRNAs regulate CD4^+^ T cells to differentiate toward various T cell subtypes including Th17 cells. In this review, we discuss the possible mechanisms of miRNAs in MS pathophysiology by regulating CD4^+^ T cell differentiation into Th17 cells, and potential miRNA targets for current disease-modifying treatments.

## Introduction

Multiple sclerosis (MS) is an autoimmune disease characterized by chronic inflammatory demyelination in the central nervous system (CNS), which can result in cognitive decline and permanent disability among young adults. The etiology of MS has been widely studied, including virus infection, genetic predisposition, lack of vitamin D, occupational exposure, and toxins. It is accepted that MS is an inflammatory and neurodegenerative disease primarily driven by myelin-reactive CD4^+^ T helper 1 (Th1) cells, CD4^+^ T helper 17 (Th17) cells, CD8^+^ T cells, and B cells that target and damage the myelin sheath. CD4^+^ Th1 cells and CD4^+^ Th17 cells are the two subtypes of CD4^+^ T cells which have been intensively investigated in MS and its animal model, experimental autoimmune encephalomyelitis (EAE). In animal studies, adoptive transfer of myelin-specific Th1 cells into naïve recipient mice was sufficient to induce features of EAE (1). Th17 cells are a newly found player in the pathology of MS and EAE model. It has been shown that the proportion of Th17 cells in peripheral blood and interleukin (IL)-17 levels in serum were increased among MS patients (2). IL-17^+^-producing T cells were elevated in the active rather than in inactive areas of MS lesions, and significantly higher densities presented within acute lesions and active borders of chronic active lesions than in normal-appearing white matter (3). This suggested that Th17 cells and IL-17 involve in the MS pathogenesis.

MicroRNAs (miRNAs) are single-stranded, approximately 22 nt non-coding RNAs that are considered as key regulators in the complex network of gene expression at the posttranscriptional level. There are approximately 24,521 miRNA loci in 206 species, which produce 30,424 mature miRNAs including more than 2,500 mature miRNAs annotated in the miRBase database (v20, June 2013) (4). miRNAs are pluripotent components that participate in several biological processes including cell development and differentiation. Drosha and Dicer nucleases are the two major miRNA machinery enzymes that convert pri-miRNAs to mature miRNAs and control the production and function of mature miRNAs. Mature miRNAs loaded into the RNA-induced silencing complex directly target mRNAs, leading to target mRNA cleavage and lower protein expression through direct or indirect interference (5).

Studies have revealed that miRNAs may contribute to MS progression and responses to treatment (6). Th17 cells are characterized by expression of retinoic acid-related orphan receptor (ROR) γ and signal transducer and activator of transcription (STAT) 3. miRNAs play major roles in Th17 cell differentiation (7), particularly through the RORγ and STAT3 signal pathway. In this review, we discuss the relationships between miRNAs, Th17 cell differentiation, and MS, pointing out the pivotal roles of miRNA in the pathophysiology of MS, and miRNAs as potential targets for current disease-modifying treatments.

## Th17 Cells in MS Pathogenesis

CCD4^+^ T cells mediate adaptive immunity to various pathogens and are critical for proper immune system homeostasis and host defense. CD4^+^ T cell-mediated autoimmunity has long been known as one of the most important aspects in MS pathogenesis. Th17 cells have been suggested as a new lineage of CD4^+^ T cells, which synthesize and secrete IL-17 and IL-22 to enhance cellular immune responses to autoimmune inflammation (8). Cytokines produced by Th17 cells are most likely to be critical pathological factors in autoimmune diseases, particularly MS. An over-exuberant response against self-antigens by Th17 cells induces several common autoimmune diseases (9) including MS. There is growing evidence from both animal models and human studies that Th17 cells and IL-17 play important roles in orchestrating MS progression. The frequency of Th17 cells is elevated in the blood and cerebrospinal fluid (CSF) of patients with MS and is higher during relapses (10). Th17 cell clones generated from the CSF and peripheral blood of MS patients expressed high levels of activation markers, adhesion molecules, and co-stimulatory molecules than Th1 clones (10). Activated Th17 cells migrate through the blood–brain barrier (BBB) into MS lesions. This migration is mediated by IL-17 and IL-22 that disrupt tight junction proteins in CNS endothelial cells (11). Th17 cells that are specific to myelin basic protein in active MS were associated with disease activity (12). After transmigration through the BBB, Th17 cells infiltrate at a high frequency into the acute MS lesions (13).

The Th17 cell lineage is characterized by expression of RORγ and STAT3, both of which are the basis for the cytokine profile, including IL-6, IL-12, IL-17, IL-22, and tumor necrosis factor (TNF), which mediate tissue inflammation. IL-17 is a cytokine can be secreted by multiple cells, such as activated T cells, natural killer cells, and neutrophils. IL-17-secreting CD4^+^ T cells (Th17 cells) are critical players in the pathology of EAE and MS. Similarly, most of the pathological functions of Th17 cells have been attributed to the secretion of cytokines, such as IL-17. IL-17 has many biological functions, such as recruiting both neutrophils and monocytes, regulating innate immunity, enhancing B cell functions, and regulating the release of pro-inflammatory cytokines including TNF and IL-1β (14). Furthermore, high levels of IL-17 exist in both serum and peripheral blood mononuclear cells (PBMCs) of MS patients (15). *In vitro* study, Th17 cells could be induced by two different conditions from naïve CD4^+^ T cells. One subset considered as non-pathogenic Th17 cells, was generated in the presence of TGF-β plus IL-6, which could abrogate Th17 cell-mediated pathology (16); the other subset was generated by IL-1β, IL-6, IL-23, and TGF-β, which was considered as pathogenic Th17 cells (17).

## Dysregulated miRNAs in MS

MicroRNAs are an emerging group of promising biomarkers in various autoimmune diseases because of their small size and stable structure in body fluids. Studying the relationships between miRNAs and MS has been a hot topic in recent years. Growing evidence shows that miRNA expression profiles might facilitate identifying the different patterns of clinical progression of MS (18).

### miRNA Profiling of Human Body Fluids

Many kinds of body fluids, such as blood, serum, plasma, CSF, and urine, can be a source to measure the expression level of miRNAs. The first study of circulating miRNA in plasma was performed by Siegel et al., revealing significant involvement of miRNAs in MS and suggesting that miRNAs may serve as potential prognostic and diagnostic biomarkers for MS (19). This study used microarray analysis to identify six plasma miRNAs, miR-614, miR-572, miR-648, miR-1826, miR-422a, and miR-22, which were significantly upregulated, and miR-1979 that was significantly downregulated in MS patients (19). miR-92a-1 was differentially expressed in relapsing–remitting MS (RRMS) versus secondary progressive MS (SPMS) and RRMS versus healthy controls (HCs). It was also associated with the expanded disability status scale and disease duration. The Let-7 family of miRNAs differentiated SPMS from HCs and RRMS from SPMS, miR-454 differentiated RRMS from SPMS, and miR-145 differentiated RRMS from HCs and RRMS from SPMS (19, 20). Other studies employed real-time RT-PCR and found higher expression of miR-155 in serum (21), and miR-141 and miR-200a in CD4^+^ T cells of MS patients in relapse than in remission (22). In addition, miR-141 and miR-200a may take part in promoting Th17 cell differentiation while inhibiting regulatory T (Treg) cells (22). miR-155 promotes T cell-driven inflammation by targeting heme oxygenase 1 (23). Using next-generation sequencing (NGS) and microarray analysis to test whole blood from MS patients, Keller et al. found that 16 miRNAs were downregulated and 22 miRNAs were upregulated in clinical isolation syndrome and RRMS. Five miRNAs were downregulated, and three miRNAs were upregulated as confirmed by microarray analysis. miR-16-2-3p was significantly upregulated, and miR-20a-5p and miR-7-1-3p were downregulated as measured by both methods (24). Compared with another study using microarray analysis, 26 miRNAs were downregulated, and 1 was upregulated in whole blood of MS patients. The downregulated group of miRNAs was found in all subtypes of MS. miR-17 and miR-20a, which were significantly under-expressed in MS, are regulators of genes involved in T cell activation (25). Sondergaard et al. investigated the expression of miRNAs in PBMCs as well as plasma and serum samples from RRMS patients by microarray analysis and identified miR-145, miR-660, and miR-939 as significantly and differentially distributed in plasma of RRMS patients compared with HCs (20).

To classify the possible function of deregulated miRNAs in target cells, many peripheral leukocyte subgroups have been isolated and examined. In a microarray analysis, 21 miRNAs had decreased expression, and 20 of them were shown to affect the expression of their target genes that are involved in the immune system (26). Studies using NGS to obtain miRNA expression profiles in a pilot cohort study of SPMS found that 97% of miRNA candidates were downregulated and 42 miRNAs were dysregulated in CD4^+^ T cells. Five miRNAs (miR-21-5p, miR-26b-5p, miR-29b-3p, miR-142-3p, and miR-155-5p) were significantly downregulated and confirmed by TaqMan assays, which targeted suppressor of cytokine signaling 6 that negatively regulates T cell activation (27). Another study using microarray analysis revealed increases of miR-128 and miR-27b in naïve CD4^+^ T cells and miR-340 in memory CD4^+^ T cells from patients with MS (28).

Compared with peripheral blood, CSF is more ideal to monitor CNS disease activity because of its close proximity to lesions, particularly the MS nidus. However, biomarkers in CSF are limited because a lumbar puncture is a traumatic procedure. Through global miRNA profiling, Haghikia et al. quantitatively confirmed that miR-922, miR-181c, and miR-633 in the CSF are differentially regulated in patients with MS (29) (Table 1). Another study demonstrated that miR-150 was elevated in MS and associated with markers of inflammation in CSF, such as the presence of oligoclonal bands, CSF cell counts, immunoglobulin G index, and candidate protein biomarkers C-X-C motif chemokine 13, matrix metallopeptidase 9, and osteopontin. This trend would be reversed after 12 months of treatment by natalizumab (30).

**Table 1:** MicroRNAs (miRNAs) involved in T helper 17 (Th17) cells development in multiple sclerosis (MS) and the experimental autoimmune encephalomyelitis (EAE) model.

miRNA	Expression change	Target	Function	Reference
miR-155-3p	Upregulated in CD4^+^ T cells in EAE mice compared with naïve mice	Dnaja2 and Dnajb1	Promote pathogenic Th17 differentiation	(48)
miR-21	Upregulated in non-pathogenic Th17 cells compared with Th1, Th2, and regulatory T (Treg) cells, induced in polarizing conditions	SMAD-7	Promote non-pathogenic Th17 differentiation	(45)
miR-17-92 cluster	miR-17-5p was upregulated in CD4^+^ T cells from MS patients compared with healthy individuals	PTEN and Ikaros family zinc finger 4	Promote pathogenic Th17 differentiation	(55, 67)
miR-183C	Highly expressed in pathogenic Th17 cells compared with other Th subsets	Foxo1	Promote pathogenic Th17 pathogenicity	(52)
miR-155	Significantly higher in sera of MS patients during relapse than MS patients during remission and healthy individuals	Ets-1	Promoted Th17 and Th1 differentiation during the induction phase of EAE	(21, 47)
miR-212	Upregulated depends on aryl hydrocarbon receptor under Th17-polarizing conditions in naïve T cells from healthy mice compared with aryl hydrocarbon receptor knockout mice	Bcl6	Promote non-pathogenic Th17 differentiation	(60)
miR-301a	Upregulated in *ex vivo* Th17 subset compared with Th1, Th2, and naïve T-helper cells	PIAS3	Promote pathogenic Th17 differentiation	(51)
miR-326	Higher in Th17 cells compared with Th1, Th2, and Treg cells in relapsing–remitting MS (RRMS) patients	Ets-1	Promote non-pathogenic Th17 differentiation *in vitro* and general Th17 *in vivo*	(39)
Let-7e	Upregulated in encephalitogenic CD4^+^ cells from EAE mice compared with CD8^+^ T cells and non-T cells	Interleukin (IL)-10	Enhance IL-17 and interferon (IFN)-γ production in the encephalitogenic CD4^+^ T cells	(40)
miR-141 and miR-200a	Both upregulated in CD4^+^ T cells of MS patients during relapsing phase compared with remitting phase and control groups	SMAD2, GATA3, and FOXO3 in relapsing phase of MS	Probably through induce the differentiation of Th17 and inhibiting differentiation to Treg cell in MS patients	(22)
miR-223	Upregulated in CD4^+^ and CD11b^+^ cells isolated from spleens of EAE models compared with healthy control (HC) mice	Roquin	Probably enhancing DC cell activation and subsequently promote Th1 and Th17 differentiations	(38, 49)
miR-26a	Significantly lower in PBLs of patients with RRMS compared with HCs, and lower expression in brain tissues from EAE mice	IL-6	Suppress Th17 differentiation and upregulate Treg function during EAE	(83)
miR-20b	Decreased in CD4^+^ T cells and significantly downregulated in non-pathogenic Th17 cells during EAE compared with neutral-treated cells	Related orphan receptor (ROR) γt and signal transducer and activator of transcription 3	Suppress non-pathogenic Th17 differentiation	(44)
miR-30a	Decreased in CD4^+^ T cells in MS patients compared with HCs, and in pathogenic Th17 cells compared with naïve T cells	IL-21R	Suppress pathogenic Th17 differentiation	(56)
miR-146a	Upregulated in CD4^+^ T cells during EAE compared with mice before EAE induction	TRAF6 and IRAK1	Suppress general Th17 differentiation	(7)
miR-15b	Downregulated in CD4^+^ T cells but not in the CD8^+^ T cells or non-T cells of MS patients	OGT	Suppress pathogenic Th17 differentiation	(43)
miR-30a	Decreased in general Th17 cells from MS patients and EAE animal models compared with naïve CD4^+^ T cells and Treg cells	IRF4	Suppress Th17 differentiation *in vitro* and during EAE	(17)
miR-132	Downregulated in CD4^+^ cells from EAE mice compared with naïve control	AChE	Decrease the secretion of IL-17 and IFN-γ and suppressed T cell proliferation	(61)

### miRNA Profiling of Lesions in Human and Animal Model

Brain-resident cells inside MS lesions may be more representative of immunological changes in MS patients. This type of study may provide important and new insights into pathological hallmarks and reveal potential targets for therapy. Junker et al. obtained miRNA profiles of active and inactive MS lesions using laser capture microdissection to isolate single cells for *in vitro* culture. As a result, 20 miRNAs were at least twice as abundant in active lesions and 22 miRNAs were at least twice as abundant in inactive lesions. miR-34a, miR-155, and miR-326, which were upregulated in active MS lesions, targeted the 3′-untranslated region of CD47 to reduce CD47 expression in brain-resident cells, particularly miR-155 (31). CD47, a ubiquitously expressed membrane glycoprotein, is abundantly expressed on phagocytic cells (32). The function of phagocytosis would be enhanced upon reduction of CD47 in macrophages. Lescher et al. analyzed miRNAs in human MS lesions together with myelin oligodendrocyte glycoprotein (MOG)_35–55_ peptide-induced EAE in C57/BL6 mice and MOG_1–125_ peptide-induced EAE in marmoset monkeys. The results demonstrated that the miRNA profiles of lesions in mice and marmoset monkeys were consistent with the miRNA profiles of active human MS lesions. miR-155, miR-326, miR-142-3p, miR-146a, miR-146b, and miR-142-5p were all significantly upregulated in active human MS lesions (33), and the miR-142-5p expression level was significantly increased in normal frontal white matter of MS patients, which was proven by upregulation of miR-142a-5p in the lumbar spinal cord at peak and post-peak phases of EAE, together with miR-142a-3p (34). However, miR-181a and miR-181-b levels in brain white matter from MS patients are downregulated (35).

Pathological and autopsy samples from patients are a valuable source that can reflect real pathological changes induced by a certain disease. In the case of MS, studies of brain tissue, circulating leukocytes, and fluids have shown altered expression of various miRNAs related to disease progression. miRNA biomarkers screened in specimens derived from MS lesions are more promising to represent the disease process, inflammatory cell motility, and/or therapeutic responses.

## miRNAs Mediate Th17 Cell Differentiation in MS and the EAE Model

**Figure 1:**
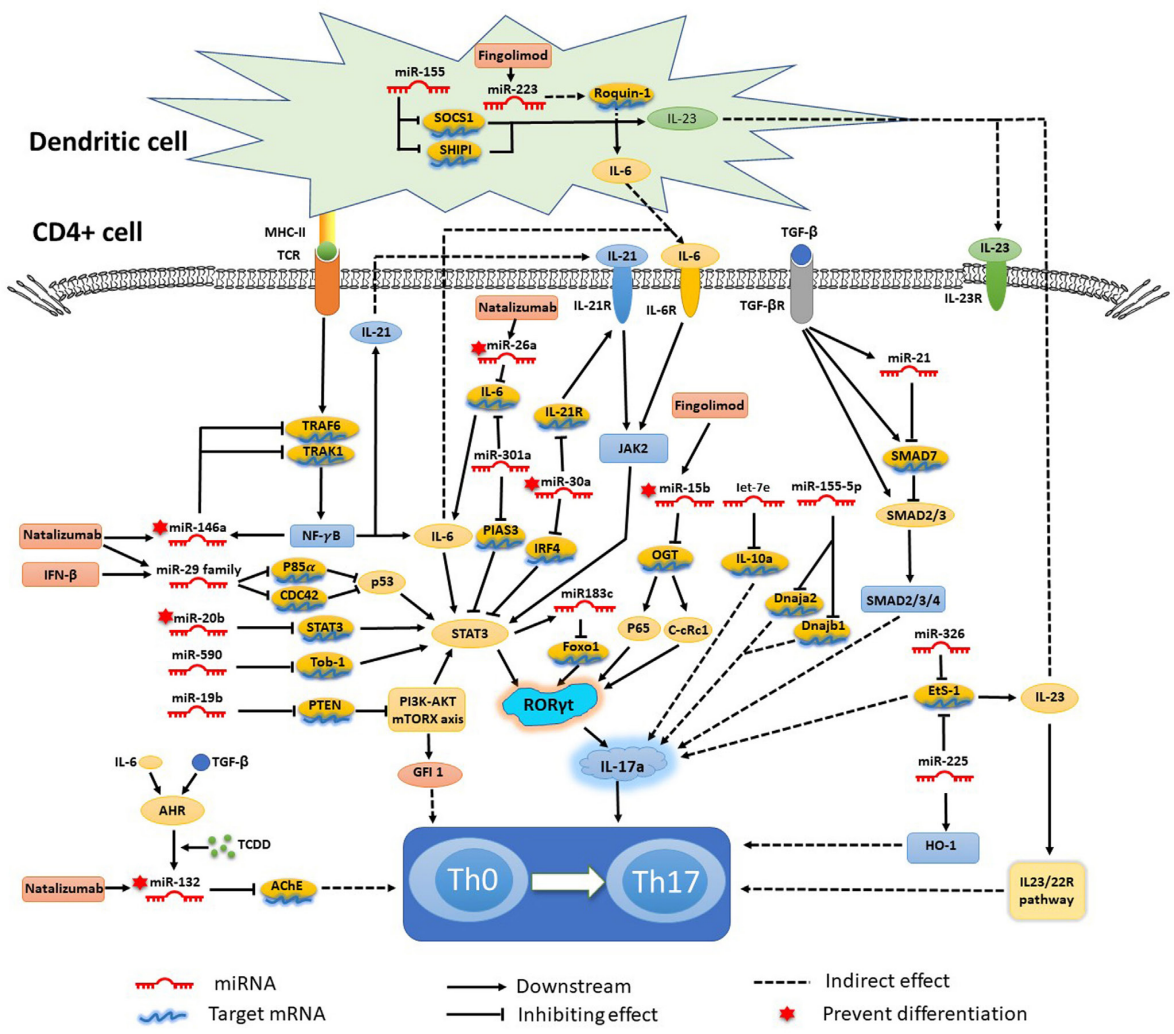
Mechanisms of specific microRNAs (miRNAs) in T helper 17 (Th17) cell differentiation and targets for current treatments. Most miRNAs promote Th17 cell differentiation and exacerbate multiple sclerosis and experimental autoimmune encephalomyelitis, while miRNA-15b, -20b, -26a, -30a, -132, and -146a show negative regulation of Th17 cell differentiation. The signal transducer and activator of transcription (STAT) 3-related orphan receptor (ROR) γt–interleukin (IL)-17 signaling pathway is downstream of most miRNAs.

As mentioned earlier, various miRNAs are related to the complex biological networks of MS. Investigations of miRNAs have found upregulation of miR-29b (36), miR-141, miR-200a (22), miR-155 (37), miR-223 (38), miR-326 (39), let-7e (40), and miR-448 (41), and significant downregulation of miR-15a/16-1 (42) and miR-15b (43) in CD4^+^ T cells of MS patients and EAE models. miR-20b (44) was decreased, while miR-21 (45) and miR-590 (46) were increased significantly in Th17 cells compared with Th1, Th2, and inducible Treg cells. *In vivo* and/or *in vitro* studies had demonstrated that most of these miRNAs mediated Th17 cell differentiation. The mechanisms of miRNAs in Th17 cell differentiation are shown in Figure 1.

### Promotion in Th17 Cell Differentiation

Silencing or knockdown of miR-326, miR-155 (21), let-7e, and miR-21 attenuate EAE with fewer Th17 cells, while their overexpression leads to more inflammation in the CNS and severe EAE. By contrast, miR-20b shows opposing trends (44). Further study has indicated that miR-326 and miR-155 promote Th17 cell differentiation by translationally inhibiting Ets-1, a negative regulator of Th17 cell differentiation (39, 47). miR-155 and miR-223, which are confirmed to be upregulated in MS and EAE models, simultaneously promote Th17 and Th1 cell differentiation in EAE mice (21) with the requirement of optimal dendritic cell production of cytokines IL-1β, IL-6 and IL-23 (37, 38). miR-155 in Th17 cells can also cause autoimmune inflammation through the clinically relevant IL-23–IL-23R pathway (47). miR-155-3p and miR-155-5p are two key miRNAs produced by the miR-155 host gene. miR-155-3p promotes Th17 cell differentiation and autoimmune demyelination by suppressing two heat shock protein 40 genes, *Dnaja2* and *Dnajb1* (48). miR-223, a myeloid cell-specific miRNA, is specifically upregulated in spinal cords and lymphoid organs, and deficiency of miRNA-223 reduces Th17 cell infiltration into spinal cords by inhibiting dendritic cell activation (49). IL-10a, as a negative regulator of EAE by suppressing Th17 cells and promoting Th2 cells, is a selectively repressed target of let-7e (50). miR-21 is upregulated in non-pathogenic Th17 cells, which intrinsically promotes non-pathogenic Th17 cell differentiation and autoimmunity by targeting and inhibiting SMAD-7, a negative regulator of TGF-β signaling. Moreover, under-expression of miR-21 in CD4^+^ T cells leads to decreased SMAD2/3 activation and IL-2 suppression, resulting in reduced sensitivity to the effects of TGF-β in T cells (45). *In vivo* and *in vitro* studies of rodent EAE models showed that myelin antigen stimulation results in significant upregulation of miR-301a, miR-21, and miR-155 in CD4^+^ T cells (51). Using specific miRNA antagonists for *in vitro* modulation, the study revealed that miR-301a contributes to the development of the pathogenic Th17 subset by targeting PIAS3 mRNA through the IL-6/23–STAT3 pathway (51). miR-183C contains three important miRNAs (miR-183, miR-96, and miR-182), is a Dicer1-regulated miRNA and is significantly expressed in pathogenic Th17 cells. Overexpression of miR-96 specifically promotes pathological cytokine production in pathogenic Th17 cells, such as IL-17A, IL-17F, IL-22, and granulocyte-macrophage colony-stimulating factor, promoting pathological effects of Th17 cells and leading to a higher disease score in EAE models. Upregulation of miR-183C can be induced by IL-6 in Th17-polarized naïve T cells through IL-6-STAT3 signaling and suppressed by TGF-β (52). miR-448-upregulated miRNAs in CD4^+^ T cells (especially pathogenic Th17 cells) and CSF of MS patients were induced by IL-1β through the NF-κB pathway. The upregulation of miR-448 increases the expression levels of IL-17A and RORγt, favoring pathogenic Th17 cell differentiation through targeting protein tyrosine phosphatase non-receptor type 2 (41), as an anti-inflammatory player with the capacity to restrain the expression of pro-inflammatory mediators (53).

The miR-17-92 cluster, as a CD28 stimulation-dependent factor, is critical for Treg accumulation and functions during an autoimmune-mediated stress response (myelin-induced EAE models) (54). It also promotes Th17 cell differentiation and impairs induction of Treg cell differentiation. Lowing miR-17-92 expression results in anabatic EAE and failure of clinical remission (54). miR-17 and miR-19b are the two core components of the miR-17-92 cluster. miR-19b promotes non-pathogenic Th17 cell differentiation by repressing the expression of phosphatase and tensin homology (PTEN), a negative regulator of the PI3K–AKT–mTOR signaling pathway. miR-17 promotes pathogenic Th17 cell differentiation by inhibiting Ikaros family zinc finger 4 (IKZF4) (55).

### Suppression of Th17 Cell Differentiation

miR-30a is downregulated in CD4^+^ T cells in MS patients and in pathogenic Th17 cells in EAE models. Overexpression of miR-30a inhibits pathogenic Th17 cell differentiation and reduces the severity of EAE by targeting mRNAs of IL-21 receptor and IRF4 (56). miR-30a also inhibits the proliferation and invasion of prostate cancer cells by targeting mRNA of sine oculis homeobox homolog 1 (57). miR-146a has been identified as a critical regulator that reduces inflammatory gene expression (58) with the opposing function to miR-155. Both of them are highly upregulated in human MS lesions. miR-146a controls Th17 cell differentiation by targeting TRAF6 and IRAK1, partially through modulation of the T cell autocrine IL-6/IL-21 pathway (7). Studies have also shown potential involvement of STAT3 and RORγt in miR-20b-induced non-pathogenic Th17 cell suppression *in vitro*, while the *STAT3* gene has been reported as a candidate target of miR-20b (44). The orphan nuclear receptor RORγt has also been described as a key transcription factor that participates in CD4^+^ T cell differentiation toward the IL-17^+^ Th17 cell lineage. RORγt is also a potential target of NF-κB, especially c-Rel and p65, which are major factors in Th17 cell differentiation and autoimmune inflammatory disease. miR-15b is a downregulated miRNA in CD4^+^ T cells of MS patients and EAE models. miR-15b inhibits pathogenic Th17 cell differentiation by targeting its potential target, O-linked *N*-acetylglucosamine transferase (OGT), and suppresses RORγt through the NF-κB (c-Rel and p65) pathway (43). miR-132, which is a member of the miR-132/212 cluster, is highly expressed in the brain (59). The miR-132/212 cluster is upregulated by aryl hydrocarbon receptor (AHR) activation under Th17 cell-polarizing conditions and affects non-pathogenic Th17 cell differentiation (60). Activation of AHR by 2,3,7,8-tetrachlorodibenzo-pdioxin (TCCD) upregulates the expression of miR-132 in CD4^+^ cells, resulting in decreased IL-17 and interferon (IFN)-γ expression and suppressed T cell proliferation by targeting acetylcholinesterase (61).

## miRNA Responses to Disease-Modifying Treatments

Investigations of miRNA response to clinical disease-modifying treatments are valuable. Several studies have focused on expression changes of miRNAs in MS and verified that miRNA expression correlates with the treatment response in MS.

### Interferon-β

Interferon β-1b was the first disease-modifying drug recommended for MS treatment with long-term efficacy and good tolerability. IFN-β suppresses IL-23 production and increases IL-27 and IL-10 production by dendritic cells, lowers the ability to promote IL-17 expression by CD4^+^ T cells, and downregulates the expression of IL-17 and IL-10 by activated STAT3 and STAT1 (62).

Hecker et al. observed significant expression changes of miRNAs in PBMCs after 1 month of IFNβ-1b treatment. Three miRNAs (miR-29a-3p, miR-29c-3p, and miR-532-5p) were confirmed to be downregulated. In addition, the miR-29 family was associated with upregulated IFN-β-responsive genes (63). miR-29 induces apoptosis in a p53-dependent manner by directly targeting p85α and CDC42 that are negative regulators of p53 (64). p53 functions as an inflammation suppressor and is a crucial negative regulator of Th17 cell differentiation *via* the STAT3 signaling pathway. Another study also affirmed that the total expression change of miRNAs in PBMCs is markedly elevated after 3 and 6 months of IFN-β therapy compared with pre-treatment levels. miR-26a-5p, which is mainly expressed in neural tissues, has been identified as the most significantly upregulated miRNA in IFN-β-treated RRMS patients. However, the significant expression change was only found in IFNβ-responder RRMS patients after 3 months of treatment. The *DLG4* gene is a potential target of miR-26a-5p, which encodes post-synaptic density protein 95 that plays a role in the signaling mechanisms of glutamate receptors (65).

### Natalizumab

Natalizumab is a recombinant humanized immunoglobulin that blocks α4-integrin at the surface of activated T lymphocytes and other mononuclear leukocytes, preventing leukocytes from adhering to endothelial cells. It was notable that the frequency of Th17 cells increased in peripheral blood and IL-17 levels increased in serum of MS patients during natalizumab treatment and return to baseline after discontinuing natalizumab (66).

A recent study of miRNAs showed that miR-17 and miR-29 are upregulated in CD4^+^ T cells during relapse and downregulated after natalizumab treatment (67–69). miR-17 is a regulator of genes involved in T cell activation (25) and promotes Th17 cell differentiation by inhibiting IKZF4 (55). After 6 months of natalizumab therapy, miR-155 and miR-132 were upregulated in MS patients, whereas miR-146a and miR-26a were downregulated. Overexpression of miR-132 decreases IL-17 and IFN-γ expression and suppresses Th17 cell differentiation (61) by targeting acetylcholinesterase (70). Analyses of miRNAs in whole blood of MS patients also revealed significant expression changes. Let-7c and miR-125a-5p were decreased, while miR-642 was increased after 6 and 12 months of natalizumab therapy compared with the baseline. In addition, the first natalizumab infusion was sufficient to trigger the change in expression of these miRNAs. Furthermore, miR-320, miR-320b, and miR-629 were differentially and significantly expressed between progressive multifocal leukoencephalopathy and non-progressive multifocal leukoencephalopathy groups after 12 months of natalizumab therapy (71). In a longitudinal study on RRMS patients, miR-18a, miR-20b, miR-29a, and miR-103 in the blood were proved to be the most strongly upregulated miRNAs by natalizumab (72). EAE in miR-106a-363 (contain miR-20b)-deficient mice had an earlier onset of symptoms and a more severe disease course. Th17-related pro-inflammatory genes RORγt and STAT3 which were predicted as targets of miR-20b, were upregulated in the spinal cord tissue (72).

### Fingolimod

Fingolimod is a sphingosine-1-phosphate analog that inhibits the egress of lymphocytes from lymphoid tissues and their recirculation, especially CCR7-expressing CD4^+^ and CD8^+^ T cells. Inhibited IL-17 and IFN expression has been found during fingolimod treatment. Moreover, the level of Th17 cells in peripheral blood fell dramatically at 1 month of treatment, but the percentage was increased among CD4^+^ T cells at 3 months until the end of follow-up (73).

A study of the transcriptome in circulating CD4^+^ T cells after 3 months of treatment identified 890 genes that were expressed differentially, 12 of which are precursors of mature miRNAs including miR-216b, miR-142, and miR-548c (74). miRNAs (miR-15b, miR-23a, and miR-223) in serum of MS patients showed slight reduction after fingolimod treatment (75), and a significant change was found after 6 months treatment (76). miR-15b suppresses Th17 differentiation by targeting OGT and suppresses RORγt through the NF-κB pathway (43). miR-223, a myeloid cell-specific miRNA, reduces Th17 cell infiltration into spinal cords by inhibiting dendritic cell activation (38, 49).

### Hematopoietic Stem Cells Transplantation (HSCT)

In addition to the treatments demonstrated earlier, studies in recent decades found that HSCT could also have beneficial effects on MS. A recent study revealed that miR-16, miR-155, and miR-142-3p which were upregulated in CD4^+^ and CD8^+^ T cells, were significantly downregulated after autologous HSCT in MS patients. Meanwhile the expressions of these miRNAs returned to normal levels at 6 months and remained stable to the end of the follow-up (77). miR-16 and miR-142-3p had been reported in regulating Treg cells activity, while miR-155 was a positive Th17 cell differentiation regulator by inhibiting Ets-1, a negative regulator of Th17 cells differentiation (39, 47).

## Advances in miRNA Techniques

We acknowledge different research groups may report different expression patterns of each miRNA even in the same disease stage and same tissues, that mainly due to different techniques for miRNA analysis. The RNA-seq and qRT-PCR are the two main methods for the expression pattern analysis for miRNAs. RNA-seq is good for large-scale analysis and occasionally used to identify novel ncRNAs including miRNAs. qRT-PCR was mostly used for the given miRNA analysis, and nowadays, several PCR-array kits were developed for large-scale known miRNA analysis in human, rat or mouse. Besides these two common methods, several new-developed methods were reviewed by Kalogianni et al. (78), such as hybridization chain reaction, target recycling, rolling circle amplification for signal enhancement, target amplification, and several sensing strategies without nucleic acid amplification. Taken together, in addition to the analysis methods, the absence of reproducibility and clear miRNA pattern identified in studies may be owing to differences in MS ethnic populations from different countries, disease status, ages, and genders.

To date, most studies are based on a single cell type, and the regulation of miRNAs among different cell types is still unknown. Recent studies indicated that extracellular vesicles including exosomes, microvesicles, and apoptotic bodies are bioactive vesicles working as miRNA carriers released by many living cells, of which, exosomes are smaller (30–100 nm) and originated from endosomal vesicles through secretion from intracellular luminal space, while microvesicles (100–1,000 nm, from activated or apoptotic eukaryotic cells) and apoptotic bodies (1–5 µm, from late stage of apoptotic cells) are formed by extensive plasma membrane budding. All these extracellular vesicles may serve as novel mediators for intracellular communication (79). Besides theses natural miRNA carriers, several man-made carriers were developed to transfer miRNAs into different cells or tissues to studying the function of miRNAs or for therapeutical uses. Because of the high molecular weight, low stability, negative charge, and high structural stiffness, it is difficult to transport miRNAs into the cytoplasm. Several methods were developed including liposomes, solid lipid nanoparticles, nanostructured lipid carriers, polymer-based nanoparticles, etc. (80). To address the limitations of polymeric and lipid-based nanoparticles, lipid–polymer hybrid nanoparticles have been developed (80). In addition, high-density lipoprotein was also used as miRNA carriers as it was used as endogenous vehicle for the transportation and metabolism of many different bioactive molecules including miRNAs (80). Furthermore, aptamer, peptide, antibody, and folate were used to combine with abovementioned methods for targeting miRNA delivery (80). Another important miRNAs delivering system is viral vectors. Now, there are several virus systems were developed for genes or ncRNAs (including miRNAs) delivery, such as lentivirus, retrovirus, adenovirus, adeno-associated virus, etc. Also, the abovementioned delivery system can be used to target or silence miRNAs expression, which is also very important for targeting endogenous miRNAs and identifying the role of miRNA or for the therapeutic uses. For miRNA silencing, one way is using oligonucleotides delivered by the abovementioned methods to inhibit miRNAs. Several chemical modification, such as antagomirs, 2′-MOEs, LNAs, and 2′-F/MOEs, were used to keep the stability of single-strand miRNA inhibitors (81). In addition, several small molecules were identified to inhibit miRNA function, such as polylysine and trypaflavine, which usually functioned as a inhibitor in miRNA-processing pathway (82). Another way to inhibit miRNA function is using viral vector to express multiple miRNA target mimics, which could bind endogenous miRNAs and left few miRNAs to bind their real targets.

## Perspectives and Conclusion

In all, miRNAs have been shown to be key regulators in mediating CD4^+^ T cell differentiation toward Th17 cells, mostly through the STAT3 signaling pathway. Some miRNAs may be biomarkers and therapeutic targets in the diagnosis/prognosis and treatment of disease activity and progression. Modulating the expression of miRNAs by specific drugs might result in fewer Th17 cells or even inhibition of the functions of pathological Th17 cells, which would be a promising anti-inflammatory treatment for MS. Several methods have been developed to regulate the level of miRNAs in tissues or cells, which hold the opportunity for disease treatment by targeting the dysregulated miRNAs. Further study may focus on miRNAs involved in cell-to-cell communication, and a future challenge will be to characterize such communication between different cell types and the possible regulatory mechanisms of miRNAs in the whole immune system.

## Author Contributions

CC reviewed the literature and was mainly responsible for writing the manuscript; YZ, JW, YY, and LP assisted in the conception of this review and figure preparation. WQ is the corresponding author and critically revised the manuscript.

## Conflict of Interest Statement

The authors declare that the research was conducted in the absence of any commercial or financial relationships that could be construed as a potential conflict of interest.
